# Social media content analysis for nutraceuticals and glaucoma

**DOI:** 10.22336/rjo.2024.48

**Published:** 2024

**Authors:** Uday Pratap Singh Parmar, Parul Ichhpujani, Vishal Abhimutt Mahesh, Suresh Kumar

**Affiliations:** Department of Ophthalmology, Government Medical College and Hospital, Chandigarh, India

**Keywords:** glaucoma, Google, nutritional supplements, nutraceuticals, social media, YouTube

## Abstract

**Aim:**

Google and various social media platforms have content on the therapeutic potential of nutritional supplements for glaucoma, but whether that information is evidence-based has not been analyzed. The current study explores such content for its quality.

**Methodology:**

Criteria of search used were “glaucoma” and “vitamins” or “nutraceuticals” or “nutritional supplements”. The first 30 search results on Google for every keyword combination were determined. The top 30 video results on Facebook Watch and YouTube for each keyword combination were selected. The initial 30 posts from Reddit and the top 30 Images on Google Images related to the keyword combination were also examined.

**Results:**

Sixty-eight websites on Google, 75 Images from Google, 39 YouTube videos, 12 video results from Facebook Watch, and 19 posts from Reddit were identified and assessed for quality.

The average Sandvik scores were 10.86 ± 2.6 (Google webpages), 10.08 ± 1.9 (YouTube videos), 10.62 ± 1.6 (Facebook Watch), and 10.26 ± 2.8 (Posts from Reddit). The average Risk Scores were 0.67 ± 0.9 (videos from YouTube), 0.49 ± 0.8 (webpages on Google), 0.33 ± 0.5 (videos from Facebook Watch), and 0.26 ± 0.5 (Reddit). The mean HON code scores were 5.15 ± 1.5 (YouTube), 6 ± 1.7 (Google webpages), 4.42 ± 1.1 (Facebook Watch), and 3.47 ± 1.8 (Reddit).

**Discussion:**

Many patients who seek information online do not consult their physicians to verify the accuracy of their search results. Thus, with this changing trend, video and online medical content analysis has attracted interest. Search engines and social media platforms may serve as adjuncts for patient counseling in current care models by providing an online educational community. Compared to non-healthcare professionals, the healthcare professionals’ information regarding nutraceuticals/nutritional supplements in glaucoma is of higher quality. Most HCPs do not recommend the use of dietary supplements as a complementary treatment for glaucoma, either because of inconclusive/insufficient data or due to contrasting studies that contradict each other. However, literature is building up with each passing day, to support nutritional supplementation as an integrative IOP-independent strategy for glaucoma management.

**Conclusion:**

The information provided by healthcare professionals is superior to that offered by non-healthcare professionals. Most HCPs advise against the use of nutritional supplements as an adjunct therapy for glaucoma, either because of inconclusive data or due to contrasting studies that contradict each other.

## Introduction

Glaucoma is a major ocular disorder characterized by neurodegeneration and progressive optic neuropathy secondary to ganglion cell degeneration in the retina and eventually, irreversible blindness [1].

Current treatment options have been limited to medical, laser, and/or surgical therapy for reducing intraocular pressure (IOP). However, glaucoma can develop even with low or normal IOP and progress despite controlled IOP. Nutritional support and alternative and complementary medicines incorporating dietary habits and lifestyle changes could affect certain underlying mechanisms associated with glaucoma onset and advancement [2].

The Internet is full of content showing the therapeutic potential of nutritional supplements/nutraceuticals, but whether that information is evidence-based and authentic needs to be further explored. This study was intended to explore the content on common web portals and social media platforms.

## Materials and Methods

### Study design and search strategy

In this cross-sectional, prospective study, four widely used digital social platforms, Google, YouTube, Facebook Watch, and Reddit, were independently searched by 2 reviewers on the 26th of March 2024 employing the keywords “glaucoma” and “vitamins” or “nutraceuticals” or “nutritional supplements”.

Google is the largest search engine, and YouTube is a leading video-based search engine favored globally for acquiring medical information [3]. Facebook Watch is a video-on-demand service managed by Meta Platforms. It offers categorized content bundles as well as customized video recommendations. Reddit is a website for rating web content, aggregating social news, and facilitating discussions. Registered members submit content such as text posts, links, videos, and images to the site. Posts are organized into user-created boards called “communities” or “subreddits”. The content is then up or downvoted by the users.

The first 30 searches on Google related to each keyword combination were noted. On Facebook Watch and YouTube, the top 30 videos for each keyword combination were selected and similarly on Reddit, the first 30 posts for each keyword combination were identified. The top 30 images from Google Images that related to each search criteria were also included for analysis. The standard search preferences of the social media platforms remained unchanged, and the searches were performed without logging in. The URLs were recorded for subsequent evaluation and quality assessment.

### Inclusion Criteria

From the social media content initially identified, all the websites, and English-language content, including videos and posts, were part of the study’s data set.

### Exclusion Criteria

Duplicates were removed from the searches: 16 for Google websites, 4 for YouTube videos, 6 for Facebook Watch videos, 7 for Google Images, and 16 for Reddit posts. Additionally, non-English-language videos were excluded (7 for YouTube and 19 for Facebook Watch). Content unrelated to the use of nutritional supplements for glaucoma was also excluded, including 6 Google websites, 40 videos from YouTube, 53 videos from Facebook Watch, and 55 posts from Reddit.

**Fig. 1:**
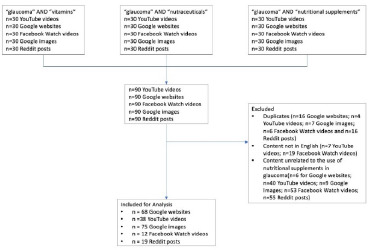
Consort Diagram

The analysis incorporated data encompassing 68 Google webpages, 75 Google Images, 39 YouTube videos, 19 Reddit posts, and 12 Facebook Watch videos. **Fig. 1** presents the CONSORT diagram.

### Content Characteristics YouTube and Facebook Watch (the video platforms)

View count, elapsed time since posting (from when the video was posted to March 26, 2024), number of likes, dislikes (if available), comments, and video length were recorded. Uploaders were categorized as academic institutions, patient/patient support groups, trading companies, research portals (such as PubMed, ResearchGate, MDPI, ARVO, GALE ACADEMIC), home-remedy /health-lifestyle based channels, solitary ophthalmologist, optometrist and non-ophthalmologist health care professionals. The videos were categorized as general discussion, information sharing, questions, personal story, research study, answer, or moderator comment. Additionally, the viewer interaction and the daily view count were calculated. The interaction index was used to measure the viewer interaction. The viewer interaction was assessed by calculating the percentage of likes (the proportion of likes to views, multiplied by 100) and the daily viewing rate (the ratio of views to the number of days since the video was uploaded). As YouTube does not offer data on video popularity based on likes and views, the Video Power Index (VPI) was computed using the formula proposed by Erdem and Karaca: the ratio of likes multiplied by the ratio of views, divided by 100 [4].

### Google web pages and Reddit posts

Uploaders were classified as research portals (such as PubMed, ResearchGate, MDPI, and GALE ACADEMIC), academic institutions, patient and patient support groups, trading companies, home-remedy or health-lifestyle-based sources, solitary ophthalmologists, optometrists, and nonophthalmologist HCPs. The categories in which the posts and websites were divided were general discussion, information sharing, personal story, research study, question, answer, or moderator comment. The study also included recording the number of comments and upvotes on Reddit posts.

### Google images

The classification of uploaders included research portals (e.g., PubMed, ResearchGate, MDPI, ARVO, GALE ACADEMIC), academic institutions, patient or patient support groups, trading companies, home-remedy or health-lifestyle-based websites, solitary ophthalmologists, optometrists, and nonophthalmologist HCPs. Images were further divided by their intended purpose, including advertisements, information sharing, questions, food or fruit images from which vitamins can be derived, and general images related to glaucoma. The captions or links below the Google images were sorted into categories like general discussion, information sharing, personal story, research study, moderator comment, question or answer.

### Quality Assessment

Each website [5], video, post, or image was assessed for quality using a previously validated Sandvik score, Health on Net (HON) code principle score [6], and previously established risk score.

The Sandvik scoring scale goes from 0 to 14 points, with 14 indicating optimal quality. Scores from 11 to 14 are considered excellent, 6 to 10 are classified as medium, and 0 to 5 are categorized as poor quality (**Table 1**).

**Table 1:** Sandvik score for content quality, 0 to 14 points total*

Category	Scoring
Ownership	2 = Name and type of provider clearly stated 1 = All other indications of ownership 0 = No indication of ownership
Authorship	2 = Author’s name and qualification clearly stated 1 = All other indications of authorship 0 = No indication of authorship
Source	2 = References given to scientific literature 1 = All other indications of source 0 = No indication of source
Currency	2 = Date of publication or update clearly stated on all pages 1 = All other indications of currency 0 = No indication of currency
Interactivity	2 = Clear invitation to comment or ask questions and e-mail address or a link to a form 1 = Any other e-mail address on the site 0 = No possibility for interactivity
Navigability	2 = Information easily found by following links from the homepage 1 = Information found only with difficulty by following links, search engine provided ifinformation widely scattered on site 0 = Information scattered around, no search engine
Balance	2 = Balanced information 1 = Biased in favor of own products or services 0 = Promoting only own products or services.

*Summated score: 11-14 excellent, 6-10 medium, and 0-5 poor quality.

The Health on the Net Foundation Code of Conduct (HON code) is intended to assess the credibility of online health and medical information. The HON code comprises 8 principles, which determine the information published on health and related content online. Each video was rated as 1 (adherent) or 0 (nonadherent) for each of these 8 principles (**Table 2**).

**Table 2:** HON code principles

Principle	Characteristic	Yes/No
**Authoritative**	Indicate the credentials of the authors	1/0
**Complementarity**	Support, not replace, the physician-patient relationship	1/0
**Privacy**	Respect the site visitors’ privacy and congeniality regarding any personal data submitted	1/0
**Attribution**	Cite the sources(s) of published information, data, and medical and health pages	1/0
**Justifiability**	Back up claims relating to benefits and performance	1/0
**Transparency**	Present accessible, accurate email contacts	1/0
**Financial Disclosure**	Identify funding sources	1/0
**Advertising Policy**	Clearly distinguish advertising from editorial content	1/0

The risk score evaluated the potential risk to patients through 4 questions. Each affirmative response earned 1 point, which led to a total risk score between 0 and 4, where a higher score signified an increased risk.

1. Does the site discourage the use of conventional medicine?
2. Does the site discourage adherence to the clinician’s advice?
3. Does the site provide opinions and experiences rather than factual data?
4. Does the site provide commercial details? For data analysis, each website, video, post, and image, was independently assessed by two reviewers (UPSP and VAM). The mean scores were then recorded and analyzed statistically. One observer (PI) randomly rechecked the videos to verify the accuracy of the collected data. Differences among assessors were addressed by review and consensus. Elevated scores on the Sandvik and HON codes and reduced Risk scores indicated quality information.

### Categorization

The content was analyzed based on the upload source and categorized into Health Care Professionals (HCP), which included academic institutions, individual ophthalmologists, optometrists, and nonophthalmology health professionals, and Non-Health Care Professionals (NHCP), which encompassed trading companies, patient or patient support groups, and home-remedy/health-lifestyle channels. The relationships between the groups and the quality of content uploaded were evaluated. HCPs were further divided into ophthalmology/optometry and nonophthalmology/non-optometry sources, and the content and the content quality scores were further compared.

### Statistical Analysis

The data collected in the study were assessed using R software version 4.1.2 (2021-11-01). Values were reported with a 95% confidence interval (CI). Descriptive data were expressed as percentages, mean values, standard deviations (SD), medians, and ranges. An independent t-test was employed to analyze continuous data. Spearman correlation analysis was utilized to assess the relationship between quality assessment scores. Additionally, linear regression analysis was applied to examine the correlation between video parameters and quality. A p-value of less than 0.05 was deemed statistically significant.

### Ethical Statement

No live animal or human subjects were included in this study. As the data used in the analysis were publicly available, approval from an institutional review board was not required. Previous studies analyzing social media content have also followed a similar approach.

## Results

68 Google websites, 39 YouTube videos, 19 Reddit posts, 12 Facebook Watch videos, and 75 Google Images were reviewed, evaluated for quality, and analyzed statistically.

The data from websites on Google was primarily focused on information sharing (n=33/68; 48%) followed by research papers (n=21/68; 30%), commercial advertisements (n=6/68; 11%), general discussion (n=4/68; 5.8%) and questions (n=4/68; 5.8%).

YouTube Videos were primarily information sharing (n=31/39; 79.4%), followed by general discussion (n=4/39; 10.2%), personal stories (n=2/39; 5.1%), and answers (n=2/39; 5.1%).

The primary nature of the Google Images was information sharing (n=34/75; 45,3%), followed by related pictures of fruits/foods the vitamins are derived from (n=13/75; 17.3%), commercial advertisements (n=11/75; 14.6%), general glaucoma (n=10/75; 13.3%), and questions (n=7/75; 9.3%). The captions and website links associated with the images were mainly focused on information sharing (n=55/75; 73.3%), advertisements (n=9/75; 12%), questions (n=8/75; 10.6%) and general glaucoma (n=2/75; 2.6%).

Facebook Watch content was primarily information sharing (n=7/12; 58.3%) followed by general discussion (n=3/12; 25%) and personal stories (n=2/12; 16.6%).

Reddit posts were mainly information sharing (n=9/19; 47.3%) followed by questions (n=8/19; 42.1%), personal stories (n=1/19; 5.2%) and general discussion (n=1/19; 5.2%). Since Google Images generally provided minimal textual information, it was excluded from the quality assessment.

### Uploaders YouTube videos

The videos were primarily uploaded by home-remedy/health-lifestyle channels (n=12/39; 30.7%) followed by academic institutions (n=5/39; 12.8%), solitary ophthalmologists (n=5/39; 12.8%), patient/patient support groups (n=5/39; 12.8%), optometrists (n=5/39; 12.8%), trading companies (n=4/39; 10.2%) and non-ophthalmologist HCPs (n=2/39; 5.1%).

### Google websites

The content was primarily uploaded by health-lifestyle-based websites (n=16/68; 23.5%) followed by research portals (n=14/68; 20.6%), nonophthalmologist HCPs (n=11/68; 16.1%), academic institutions (n=10/68; 14.7%), trading companies (n=7/68; 10.2%), optometrists (n=6/68; 8.8%), solitary ophthalmologists (n=3/68; 4.4%) and patient/patient support groups (n=1/68; 1.5%).

### Google Images

The images were primarily uploaded by health-lifestyle-based sources (n=19/75; 25.3%) followed by research portals (n=18/75; 24%), trading companies (n=15/75; 20%), academic institutions (n=9/75; 12%), optometrists (n=6/75; 8%), nonophthalmologist HCPs (n=5/75; 6.6%) and solitary ophthalmologists (n=2/75; 2.6%).

### Reddit posts

The posts were primarily uploaded by health-lifestyle-based sources (n=8/19; 42.1%) followed by academic institutions (n=6/19; 31.5%), patient/patient support groups (n=4/19; 21%) and solitary ophthalmologists (n=1/19; 5.2%).

### Facebook Watch

The videos were predominantly uploaded by patient and patient support groups (n=8/12; 66%) followed by solitary ophthalmologists (n=2/12; 16.6%), non-ophthalmologist HCPs (n=1/12; 8.3%), and optometrists (n=1/12; 8.3%).

### Quality assessment scores

The general characteristics of the data for YouTube, Facebook Watch, and Reddit are presented in **Table 3**.

**Table 3:** General characteristics of the data for YouTube, Facebook Watch, Reddit

Parameters	Mean ±SD	YouTube Median		Range			
**Length (in minutes)**	10.1 ±11.3	6.5		0.45		50.5	
**Number of views**	61756.5 ±177893.0	1631.0		8.00		817435.0	
**Daily view rate**	60.7 ±158.5	2.5		0.01		746.5	
**Time since upload (days)**	939.9 ±679.6	777.0		11.00		2734.0	
**Time since upload (years)**	2.6 ±1.9	2.1		0.03		7.5	
**Number of likes**	1805.7 ±5974.9	32.0		0.00		29000.0	
**Number of comments**	77.0 ±229.2	2.0		0.00		1239.0	
**Viewer interaction index**	3.4 ±3.7	2.3		0.00		16.3	
**VPI**	117.5 ±128.1	78.4		0.00		558.4	
**Facebook Watch**							
**Length (in minutes)**			2.10 ±2.9		1.24	0.22	10.5
**Number of views**			2219.80 ±3157.3		409.00	7	8900.0
**Daily view rate**			3.73 ±8.2		1.09	0.01	29.1
**Time since upload (days)**			1077.70 ±1235.1		708.00	118	4500.0
**Time since upload (years)**			2.95 ±3.4		1.94	0.32	12.3
**Number of likes**			14.75 ±13.5		12.50	0	47.0
**Number of comments**			6.25 ±12.7		1.50	0	45.0
**Viewer interaction index**			7.86 ±20.2		1.42	0	71.4
**VPI**			1183.44 ±3033.0		214.08	0	10749.4
**Reddit**							
**Comments**			3.3 ±4.0		2	0	16
**Upvotes**			6.3 ±5.4		5	1	19

The average Sandvik scores were 10.08 ± 1.9 (YouTube videos), 10.66 ± 2.6 (Google webpages), 10.92 ± 1.6 (Facebook watch), and 10.26 ± 2.8 (Reddit posts). The average Risk Scores were 0.67 ± 0.9 (YouTube), 0.49 ± 0.8 (Google websites), 0.33 ± 0.5 (Facebook videos), and 0.26 ± 0.5 (Reddit posts). The average HON code scores were 5.15 ± 1.5 (YouTube), 6 ± 1.7 (Google websites), 4.42 ± 1.1 (Facebook watch), and 3.47 ± 1.8 (Reddit posts).

**Table 4** emphasizes the significant difference (P<0.05) in all 3 quality assessment scores between the content uploaded by HCPs and NHCPs on Google and YouTube and the Sandvik and HON code scores on Reddit.

**Table 4:** Comparison of online content regarding glaucoma and nutritional supplementation across social media platforms

Scoring	YouTube			p-value
	Total (n=39) Mean ±SD	HCP (n=17) Mean ±SD	NHCP (n=22) Mean ±SD	
**Sandvik**	10.08 ±1.9	11.06 ±1.5	9.31 ±1.4	0.000
**Risk score**	0.67 ±0.9	0.29 ±0.3	0.95 ±1.0	0.000
**HON code**	5.15 ±1.5	5.9 ±1.1	4.54 ±1.3	0.000
**Google Webpages**				
	Total (n=68)	HCP (n=54)	NHCP (n=14)	
**Sandvik**	10.66 ±2.6	11.20 ±2.1	8.57 ±3.3	0.0129
**Risk score**	0.49 ±0.8	0.30 ±0.5	1.21 ±1.3	0.0175
**HON code**	6.00 ±1.7	6.41 ±1.5	4.43 ±1.7	0.0007
**Facebook Watch**				
	Total (n=12)	HCP (n=3)	NHCP (n=9)	
**Sandvik**	10.92 ±1.6	12.00 ±1.0	10.56 ±1.6	0.117
**Risk score**	0.33 ±0.5	0.33 ±0.6	0.33 ±0.5	1.000
**HON code**	4.42 ±1.1	5.68 ±0.6	4.00 ±0.9	0.117
**Reddit**				
	Total (n=19)	HCP (n=5)	NHCP (n=14)	
**Sandvik**	10.26 ±2.8	14.00 ±0.0	8.93 ±1.9	0.000
**Risk score**	0.26 ±0.5	0.20 ±0.4	0.29 ±0.5	0.730
**HON code**	3.47 ±1.8	5.80 ±0.4	2.64 ±1.3	0.000

HCP = Health Care Professionals; NHCP = Non-Health Care Professionals

No significant difference was found across the platforms when the quality assessment scores were compared between ophthalmologist/optometrist sources and non-ophthalmologist/optometrist sources among HCPs (**Table 5**).

**Table 5:** Comparison of the quality assessment parameters between ophthalmologists and non-ophthalmologists among health care professionals

Scoring	YouTube			p-value
	Total (n=17)	Ophthalmologists (n= 10)	Other HCP (n=7)	
	**Mean** ±**SD**	**Mean** ±**SD**	**Mean** ±**SD**	
**Sandvik**	11.06 ±1.5	11.00 ±1.6	11.14 ±1.1	0.863
**Risk score**	0.29 ±0.3	0.10 ±0.3	0.57 ±0.4	0.321
**HON code**	5.9 ±1.1	6.30 ±1.3	5.42 ±0.4	0.211
**Google Webpages**				
	**Total (n=54)**	**Total (n= 40)**	**Total (n=14)**	
**Sandvik**	11.20 ±2.1	11.18 ±2.2	11.29 ±1.7	0.848
**Risk score**	0.30 ±0.5	0.25 ±0.4	0.43 ±0.6	0.351
**HON code**	6.41 ±1.5	6.40 ±1.7	6.43 ±1.0	0.940

HCP = Health Care Professionals

In the linear regression analysis, a statistically significant difference was observed in the daily view count, comments, and the risk score in video content uploaded by HCPs, and between video length and the risk score (p < 0.05) in video content uploaded by NHCPs (**Table 6**).

**Table 6:** Correlation between Sandvik, Risk Score, and HON code scores for the entire social media content analyzed

	Sandvik	Risk Score	HON code
**Sandvik**	1		
**Risk Score**	R = -0.353 p = 0.0002	1	
**HON code**	R = 0.761 p = 0.000	R = -0.334 p = 0.0004	1

**Table 7** demonstrates a significant positive correlation (p < 0.000) between the Sandvik score and the HON code and a negative correlation (p < 0.000) between the Risk score with the Sandvik and HON code scores for the entire data.

**Table 7:** Linear regression analysis between countable parameters

	Video Parameters	Sandvik		Risk Score		HON Code		
		p-value	R	p-value	R	p-value		R
**HCP**	Video Length	0.531	0.025	0.465	0.034	0.122	0.1432	
	View Count	0.266	0.077	0.009	0.356	0.705	0.0092	
	Daily View Count	0.880	0.001	0.002	0.473	0.969	0.0001	
	Comments	0.873	0.002	0.003	0.423	0.987	0.0000	
**NHCP**	Video Length	0.178	0.093	0.021	0.250	0.962	0.0001	
	View Count	0.388	0.040	0.351	0.046	0.801	0.0034	
	Daily View Count	0.567	0.018	0.124	0.120	0.458	0.0294	
	Comments	0.356	0.045	0.251	0.069	0.748	0.0056	

HCP = Health Care Professionals; NHCP = Non-Health Care Professionals

## Discussion

When used appropriately, Google and YouTube are valuable sources that could improve the understanding and learning experience of the public and medical professionals. The upside of search engines and social media platforms is that medical information can be distributed to many viewers [7]. However, these valuable assets can become dangerous liabilities because of misleading, sometimes harmful unfiltered videos. Thus, assessing the accuracy and quality of health-related content is crucial. Many patients depend on health-related information found online, and, sometimes, use it as a substitute for advice or consultations from their physicians. Many patients who seek information online do not consult their physicians to verify the accuracy of their search results. Thus, with this changing trend, video and online medical content analysis has attracted interest. Oydanich et al. showed that YouTube videos on information about glaucoma had adequate quality and reliability scores [8]. Jia JS recently described the quality and characteristics of most internet search results concerning medical marijuana and glaucoma [9]. We undertook the current study to find out the character of the available information on nutraceuticals for glaucoma.

### Google

Among the 54 HCP sources on the Google search engine 45 (83.3%) recommended against the use of vitamins as a treatment for glaucoma, the reasons being either the presence of inconclusive/insufficient data, the presence of contrasting studies that contradict each other, studies done on smaller cohorts or the last clinical trials done on humans. Some of these sources agreed on a potential association between the supplements and the pathogenesis/progression/prognosis of glaucoma based on the conducted research. However, more literature and clinical trials are needed before prescribing these as prophylactic or therapeutic agents in patients at risk of, or with already diagnosed glaucoma.

Six (11.11%) sources did not conclude. These included a general discussion on vitamins and eye health, ongoing studies/research papers, and questions posed by patient support groups or other HCPs.

Three (5.55%) sources were pro vitamins/supplement use in glaucoma. These included a research abstract concluding with a statistically significant association between dietary flavonoids and an IOP decrease in patients with glaucoma, a solitary ophthalmologist recommending ginkgo biloba use, and an optometry website promoting a Vitamin B12 supplement.

Among the 14 NHCP sources, 10 (71.4%) were pro vitamin/supplement use, notably 7 (50%) of these were commercial websites promoting their supplements (Dr Whitakers™, Metrogenol Ad™, ALCOMA™, Optic nerve formula™, FitEyes™, and, Eye Promise™).

Three slideshows were uploaded by the website SlideShare but the credentials of the presenters or the references for their data were not mentioned.

The nutritional supplements encountered while reviewing the content on Google included Vitamins (A, B1, B3, B12, D, E), antioxidants (Gingko Biloba, bilberry [anthocyanins], Zinc), forskolin, Triphala (an Indian Bengali herb), dietary flavonoids, black currant, topical coenzyme Q, citicoline, Lisosan G, lutein, zeaxanthin, and omega 3 fatty acids.

While most websites discussed multiple vitamins and nutritional supplements in their association with glaucoma, 2 sources focused on specific associations citing indexed research articles namely that of Vitamin D and Lisosan G.

### YouTube

Among the 17 videos uploaded by HCPs, only 5 (35.7%) videos supported vitamins/supplements use in glaucoma. These included 2 videos on ginkgo biloba referencing already published literature about the same, one about the benefit of organic pasteurized eggs that are rich in lutein and their role in decreasing glaucoma risk, one about vitamin B12 being proven beneficial in normal tension glaucoma by an ophthalmology hospital from India, and 1 about bilberry extracts citing a research study. However, one video established that the association between the antioxidant properties of vitamin C and its role as a neuroprotectant in glaucoma was against therapeutic recommendation due to the higher concentrations required for potential antiglaucoma effect and the subsequent risk of side effects.

Among the five most-watched YouTube videos, three were uploaded by healthcare professionals, while the remaining two were posted by a nutraceutical company and a home remedy channel, respectively.

Most uploaders (n=20/22; 90.9%) under NHCPs advocated vitamin/nutritional supplementation use in glaucoma. These primarily included home-remedy/health-lifestyle-based channels (n=12) promoting vitamin-rich natural foods (carrots, spinach, kale, turmeric, and other herbs) or supplements like Goji berry, wolfberry, blueberry, beta carotene, and AC Carbamide. Commercial vitamins like Dr Whitakers™, VISOFAR™, MULTIVIMIN™, Netsurf Nutraceuticals™, Renatus Nova,™ and Vermilon TCM™ herbs were also promoted by the channels of trading companies or by health-lifestyle-based ones.

### Google Images

Information sharing was the primary (n=34/75; 45,35) character/purpose of the Google images analyzed. These images included flow charts, tables, and animations analyzing the various vitamins and nutritional supplements and their proposed role in glaucoma prevention, pathogenesis, or progression. This was followed by images of fruits, vegetables, fish, and other natural foods (n=13; 17.3%). Although these images had no text, their captions or the corresponding websites explained their relationship with the keywords used. The third most common type of images were commercial ones (n=11; 14.6%) promoting vitamins and nutritional supplements and linking to corresponding trading or health-lifestyle-based supplements. These included VITEYES™ optic nerve support, LifeExtention B complex™, EyeBright™, Naturelo™, ALCOMA™, and a Magnesium supplement by Whitaker™ Nutrition.

### Reddit

Notably, 8 posts out of 19 (42.1%) were about the association of vitamin B3 (nicotinamide) with glaucoma. 3 of these were posted by HCP sources and 5 by NHCPS.

Among HCPs, 3 posts were specifically about vitamin B3, 2 being research studies.

The other 2 posts were about vitamin and general nutritional supplementation and glaucoma. No clinical recommendations were made.

Among the posts uploaded by NHCPs, 5 were uploaded by patients/patient support groups ranging from a general discussion about the natural treatments available for glaucoma to a personal story from a patient who used vitamin C as one. Separate posts uploaded by NHCPs recommended Vitamins (A, B3, C, B13), Pyruvate, Gingko, and Zinc use in glaucoma.

The top 5 posts on Reddit with the most upvotes were posted by a solitary ophthalmologist, 2 academic institutions, a patient support group, and a patient. A solitary ophthalmologist shared the post with the most upvotes. He shared information regarding research on vitamin B3 and glaucoma.

### Facebook Watch

Among the videos uploaded by HCPs (3/12; 25%), one was about the association between nutraceuticals and eye health in general, while the other 2 specifically talked about glaucoma and its possible association with vitamin B3 and Astaxanthin, respectively. Recommendation as a prevention or treatment was not made.

Among NHCPs, eight videos were uploaded by patient or patient support groups and the vitamins and supplements discussed included A, B3, C, E, and Zinc.

Two of these were personal stories, 1 was a patient inquiring about what vitamin he should use to treat his glaucoma naturally. The others were information and articles shared by support groups.

The top 5 videos on Facebook Watch, with the most views, were uploaded by a news channel, an ophthalmologist, an optometrist, a patient support group, and a patient. A solitary ophthalmologist uploaded the video with the most views. He talked about an OTC vital supplement (B complex) that can be useful for treating glaucoma.

So, to conclude, social media gives patients an unmonitored platform to voice their doubts, suggestions, and grievances, and to extend mutual support to others suffering from similar ailments. Online information may be helpful to physicians, such as adverse drug reactions, areas for service improvement or common topics that patients might be interested in knowing can be flagged [10,11].

Search engines and social media platforms may serve as adjuncts for patient counseling in current care models by providing an online educational community. However, the risks of misinformation need to be understood.

Compared to non-healthcare professionals, the healthcare professionals’ information regarding nutraceuticals/nutritional supplements in glaucoma is of higher quality.

Most HCPs do not recommend the use of nutritional supplements as a complementary treatment for glaucoma, either because of inconclusive/insufficient data or due to contrasting studies that contradict each other. However, literature is building up with each passing day, to support nutritional supplementation as an integrative IOP-independent strategy for glaucoma management [12].

## Conclusion

The information provided by healthcare professionals is superior to that offered by nonhealthcare professionals. Most HCPs advise against the use of nutritional supplements as an adjunct therapy for glaucoma, either because of inconclusive data or due to contrasting studies that contradict each other.
